# ALF–Score—A novel approach to build a predictive network–based walkability scoring system

**DOI:** 10.1371/journal.pone.0270098

**Published:** 2022-06-24

**Authors:** Ali M. S. Alfosool, Yuanzhu Chen, Daniel Fuller

**Affiliations:** 1 Department of Computer Science, Memorial University of Newfoundland, St. John’s, Newfoundland, Canada; 2 School of Computing, Queen’s University, Kingston, Ontario, Canada; 3 School of Human Kinetics and Recreation, Memorial University of Newfoundland, St. John’s, Newfoundland, Canada; University of Bradford, UNITED KINGDOM

## Abstract

Walkability is a term that describes various aspects of the built and social environment and has been associated with physical activity and public health. Walkability is subjective and although multiple definitions of walkability exist, there is no single agreed upon definition. Road networks are integral parts of mobility and should be an important part of walkability. However, using the road structure as nodes is not widely discussed in existing methods. Most walkability measures only provide area–based scores with low spatial resolution, have a one–size–fits–all approach, and do not consider individuals opinion. Active Living Feature Score (ALF–Score) is a network–based walkability measure that incorporates road network structures as a core component. It also utilizes user opinion to build a high–confidence ground–truth that is used in our machine learning pipeline to generate models capable of estimating walkability. We found combination of network features with road embedding and points of interest features creates a complimentary feature set enabling us to train our models with an accuracy of over 87% while maintaining a conversion consistency of over 98%. Our proposed approach outperforms existing measures by introducing a novel method to estimate walkability scores that are representative of users opinion with a high spatial resolution, for any point on the road.

## Introduction

Road networks serve as a major part of the urban fabric, connecting and dividing different parts of our cities. Most people use roads on a daily basis making road networks a crucial part of our daily lives. Mobility is defined as having access, and the ability to get to places necessary for “living a healthy life”. According to a study [1] published by Statistics Canada, 12.6 million Canadians reported in 2016 to have commuted by car to work with the average duration of the commute being 24 minutes, and the median distance to workplace being 8.7 kilometres. Furthermore, close to 1 million car commuters spent at least 60 minutes travelling to work. Trips to work, grocery store, hospital or even casual jogs and road trips mainly occur on walkable or driveable roads.

With the increase of publicly available geographic information systems, road network data is now freely accessible from numerous sources. Due to this availability, road network data can be used as a good source for various types of analysis like road importance [2–5] road characteristics [6, 7] city planning [8–10] creating walkability or bikeability scores, or examining the association between diabetes and obesity in local communities with walkability of the neighborhoods [11, 12]. Walkability is a term used to describe aspects of the built and social environment with important population level impacts on physical activity and health.

Only a small amount of work has focused on developing a conceptual definition of walkability. However, the term has been widely used in the academic literature since the late 1990s by urban planners, the general public, and researchers [13–21] when researchers began to examine the association between walkability and various factors including travel behavior, urban design, real estate, physical activity, and obesity. Although there are multiple operational definitions of walkability in the literature [22–27] there is no single agreed–upon conceptual definition of walkability. Simply put, Walkability is a way to show how walkable/connected/accessible our surroundings are, with respect to walking. Walkability is typically used by researchers as a measure to operationalize characteristics of roads combined with other characteristics to create meaningful indicators for the environment. Knowing how walkable an area is (i.e. Walkability score) is an important factor in everyone’s lives, especially with the spread of the COVID–19 restrictions and limitations to how and when we go outside.

Walkability is a crucial metric that provides an in–depth insight into the effects of one’s geographical positioning, accessibility to various criteria within a walking distance, and ability to mobilize on a regular basis over one’s health. Walkability scores provide important knowledge about the roads and neighbourhoods around us. An accurate walkability metric can significantly and positively improve the way public health professionals study, analyze, understand, and address major health issues affecting millions of people around the world, such as obesity and diabetes. An important influencing factor is the ability to produce walkability scores relevant to varying circumstances and personalized to individuals. However, current walkability measures do not consider individual preferences and are generic. Moreover, an in–depth study and analysis of walkability requires high spatial resolution scores that can be well refined over small geographical regions. Most existing measures however, are either mainly area based or provide low spatial resolution.

Our Active Living Feature Score or ALF–Score, is a network–based walkability measure that utilizes road network structure alongside user opinion and various other features through machine learning approaches to build predictive models capable of generating high spatial resolution and network–based walkability scores. Below is a list of the main contributions of this paper:

1. High Spatial Resolution—compared to existing walkability measures, such as Can–ALE [2] which provides area based walkability scores, in this paper, we show that ALF–Score is capable of producing a much higher point–level walkability resolution which establishes point–specific walkability scores for all points on the road to provide more refined and usable walkability scores.
2. Road Network Structure Incorporation—ALF–Score relies on road network structures and does not treat area–based locations as individual vectors. Roads are connected and therefore spatial correlations between various roads and points exists. Incorporating road network structure allows determination of similarities throughout the entire network regardless of physical distances, to help improve the overall accuracy of estimated scores.
3. User Opinion—most existing walkability measures are presumptive and generic. ALF–Score utilizes end-users’ opinions as an input fed to its pipeline to ensure an appropriate representation of user opinion in the estimation of the walkability of each specific point.
4. Data Scalability—The scalability of this work is twofold. First, since a small fraction of all users provide their opinion, ALF–Score takes certain measures to ensure appropriate, fair and consistent distribution of user opinions on the road network. Second, the method proposed can be applied to many cities all over the world.

In this paper, we start by reviewing related works revolving around walkability measurement. We then present our results and discuss our findings. We will then discuss various data types (publicly available, and crowd–sourced) and methods used in our pipeline with a brief overview of our crowd–sourcing platform. Additionally, we will explore our experimental setup and how we take on the problem using machine learning. Finally, we will examine the consistency and validation measures used to verify our results.

**Fig 1:**
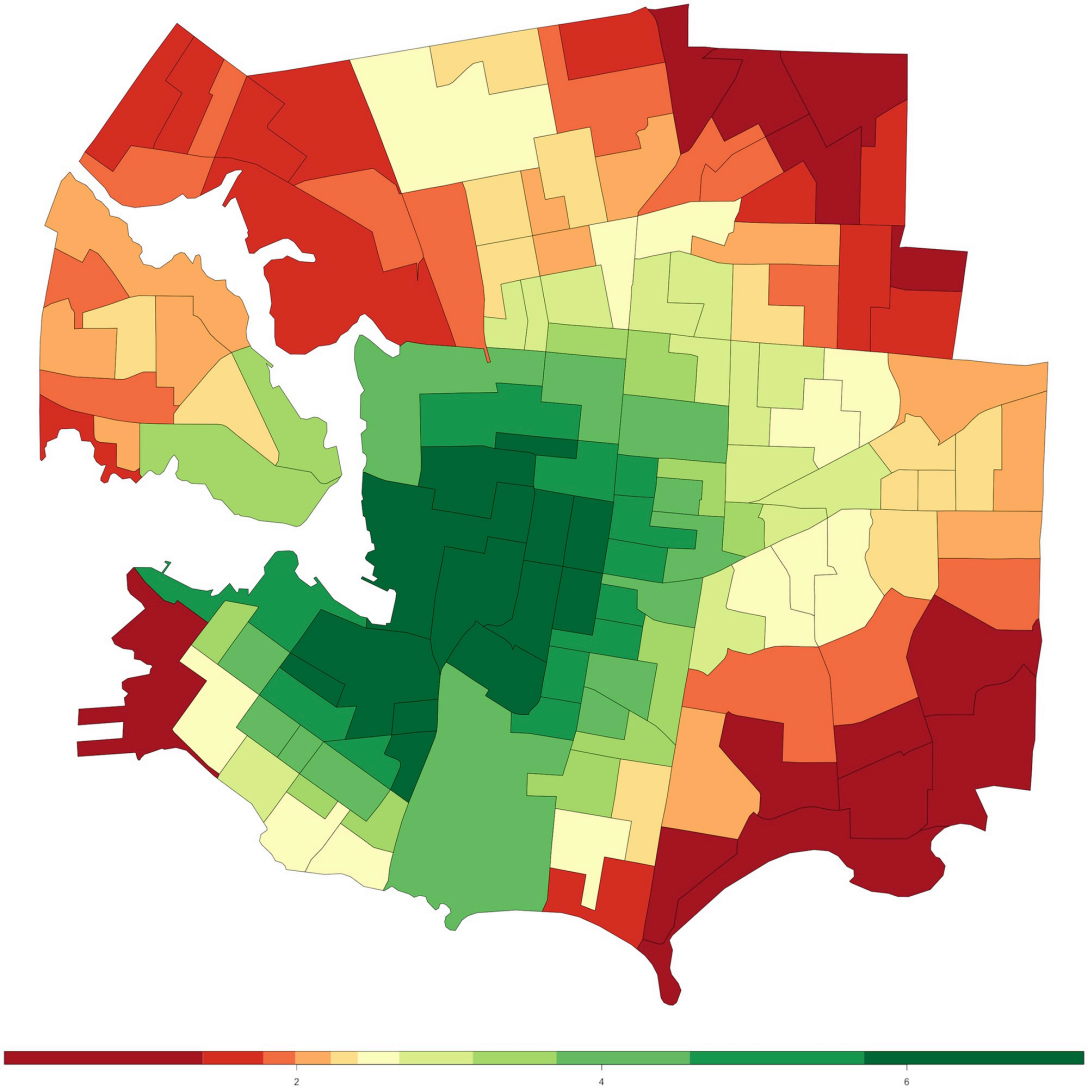
Can–ALE walkability score for Victoria, BC. Can–ALE scores are assigned by dissemination area. Dark green shows most walkable while dark red shows least walkable. (generated through RStudio [31] Version 1.2 from rstudio.com).

There are a number of existing walkability measures that provide walkability scores for Canada. For the purpose of comparison, we choose the Canadian Active Living Environments Database (Can–ALE) [2] and Walk Score [3] as they are both commonly used by researchers and end–users alike. These existing measures have some similarities to our proposed method, ALF–Score, such as the use of Points of Interests (POI) and generating walkability scores as their output. However, there are many differences among them such as the level of precision for generated scores where some of these existing measures (eg. Can–ALE) are area–based covering an area, typically a Dissemination Area, with only a single score, whereas the point–level precision found in ALF–Score provides walkability scores for any given point on the road. Furthermore, lack of machine learning processes as well as user opinion and preferences (eg. in both Can–ALE and Walk Score) are also observed among some of the other differences. Additionally, while Can–ALE is open–source, Walk Score is closed–source based on proprietary methods. These measures each have different strengths and limitations. Can–ALE is one of the most prominent and publicly available walkability scores in Canada. Fig 1 shows Can–ALE scores for Victoria, BC. Can–ALE is an area–based measure at the Dissemination Area (DA) level [28, 29]. A DA is a small, relatively stable geographic unit composed of one or more adjacent dissemination blocks and is the smallest standard geographic area for which all census data are disseminated. DAs’ physical size may vary depending on their geographical location but, they normally have a population of between 400 to 700 persons and there are approximately 54,000 DA’s in Canada. Can–ALE, widely used for walkability scores in Canada, utilizes intersection density as well as dwelling density to devise a walkscore for each Canadian DA. The measure also includes the number of POI within 1km buffer around the DA’s centroid. According to Canadian Active Living Environments Database (Can–ALE) User Manual & Technical Document [30] “Can–ALE measures are based on one–kilometre, circular (Euclidean) buffers drawn from the centre points (centroids) of dissemination areas (DAs)”. Can–ALE’s only measure of road network importance is a simple count of the number of 3 (or more)–way intersections per square kilometre of the buffer around a dissemination area’s centroid. Walk Score is another well–known walkability tool which uses a proprietary method that has a number of features including population density and road metrics such as block length and intersection density. According to its authors, they use a patented system to measure the walkability of any location and focus on analyzing hundreds of walking routes to nearby amenities. Although, since it is based on a closed–source system using proprietary methods, we do not know if the scores are actually calculated for every individual location instead of general areas that provide distributed scores down to locations within each area. Their walkability score (between 0–100) is awarded based on distance to amenities of certain categories (eg. grocery stores, coffee shops, restaurants, bars, movie theatres, schools, parks, libraries, book stores, fitness centres, drug stores, hardware stores, clothing/music stores). Amenities within a 5–minute walk (.25 miles) are given maximum points. They use a decay function to devise what score should be given to more distant amenities, where no score is given for amenities located farther than a 30–minute walk. Walk Score also measures pedestrian friendliness by analyzing population density and road metrics such as block length and intersection density. Their data sources include Google Maps, Factual, Great Schools, Open Street Map, the U.S. Census, Localeze, and places added by the Walk Score user community.

ALF–Score is a measure that provides a faster and a better walkability scoring system that aims to fill in the gap and address major limitations of existing measures by introducing use of opinion–based crowd–sourced user parameters in conjunction with road network characteristics to provide scores that better represent one’s perspective of walkable areas while utilizing road network structure to improve accuracy and spatial resolution.

Road network is a system of interconnected roads designed to accommodate vehicles, bicycles, and pedestrian traffic. According to Urban Securipedia [32], road networks consist of “a system of interconnected paved carriageways which are designed to carry buses, cars and goods vehicles”. Furthermore, road networks form the “most basic level of transport infrastructure within urban areas, and will link with all other areas, both within and beyond the boundaries of the urban area”. For computational purposes, road maps are typically converted into road network structure. Road networks are a form of complex network where nodes refer to physical geographical points and edges are the connections between two points. Road networks can be represented as graphs. We define such graphs as *G* = (*V*, *E*) where *E* represents a list of edges/links and *V* represents a list of vertices/nodes. Points in these networks represent nodes while roads and their connections where two or more nodes connect, represent edges. Adjacency matrix or adjacency list are two common ways to represent graph data structure. Since the road networks are generally sparse and can be vast, the adjacency list is often preferred.

There are a number of data types required for this work such as user opinion, road network structure, points of interest, and those features derived form the road structure such as edge list, node/edge centrality and node embedding. Since road networks are accessible through many different sources such as OpenStreetMaps (OSM) [33] and Statistics Canada [34], different data formats are also available for processing. However, for us the main concern when selecting the data source is data comprehension and accuracy. OSM follows an XML scheme containing nodes and ways elements with former defining points in space and the latter, defining linear features and area boundaries. The road network files are filtered to only include highway tags [35] encompassing only walkable and drivable roads. Points Of Interest or POI data for this research was exclusively extracted from OSM, with “amenity” key [35] being used as the most relevant set of POIs covering numerous categories such as: Sustenance, Education, Transportation, Financial, Healthcare, Entertainment, Arts & Culture, and Others.

## Materials and methods

**Fig 2:**
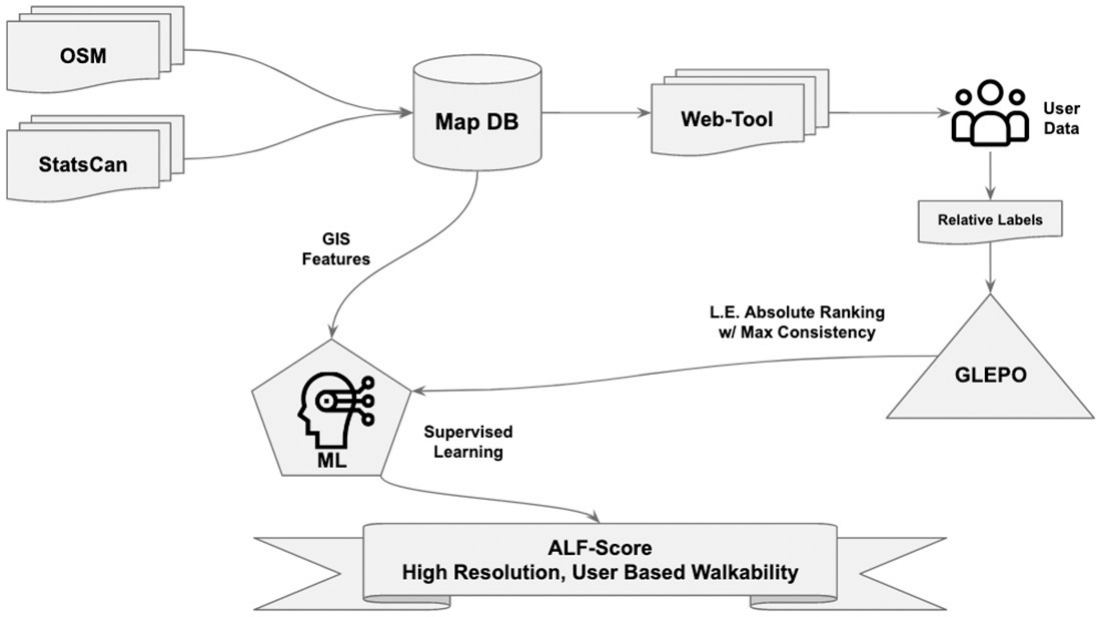
ALF–Score core pipeline. ALF–Score utilizes various GIS features such as road network structure, POI as well as features derived from road networks such as various centrality measures and road embedding. GLEPO’s linear extension of user opinions that produces a global view of relative user opinions is then aligned with the GIS features as an input of our supervised machine learning processes. Walkability estimates that are produced through trained models will have a high spatial resolution, are representative of user opinion and provide a better insight of different regions and neighbourhoods. (Figure drawn by the authors).

The overall ALF–Score pipeline (shown in Fig 2) requires various data input as well as processes. However, since ALF–Score is a network–based walkability measure, an important step in this pipeline is to better utilize road networks as well as other road characteristics. To this end, ALF–Score incorporates a map database derived from road network and POI inputs extracted from OpenStreetMap and Statistics Canada. Other GIS features such as road embedding and various centrality measures were later generated from the road network structure and used as additional road network based features. In addition, crowd–sourced user opinion is collected through our web–based data collection platform which is specifically developed for this purpose. The web tool allows collection of user opinion within groups of 5 locations, all of which only relative among their respective groups. The crowd–sourced data is then processed through GLEPO in such a manner that the relative structure of each submission is converted into a global view within all user submissions. GLEPO’s output of user ranking along side our GIS–derived features are then fed to our supervised machine learning pipeline to train predictive models that are capable of estimating walkability score for any given point on the road that falls within the map database coverage.

Participation in the crowd–sourced data collection is completely voluntarily. All participants provide their informed consent online through our online crowd–sourcing web–tool. The consent form is the first thing participants see on the web–tool. Participants are informed that their participation is voluntary. A popup appears when accessing the web–tool informing volunteer participants that by submitting the form on our online crowd–sourcing web–tool, they confirm they have fully read and understood our consent form and privacy notes and have consented to participate in this study and have their data collected and used in the research. Relevant information such as the informed consent form as well as our privacy notes are visibly and clearly available via the popup window and throughout the web–tool. Participants are notified online through the popup and footer content that user submission will automatically be considered as their consent to participate. The proposal for this research has been reviewed by the Interdisciplinary Committee on Ethics in Human Research (ICEHR) and found to be in compliance (ICEHR Approval Number: 20220406–SC) with Memorial University’s ethics policy and in accordance with the Tri–Council Policy Statement on Ethical Conduct for Research Involving Humans (TCPS2), the project has been granted full ethics clearance. This study did not include any minors.

**Fig 3:**
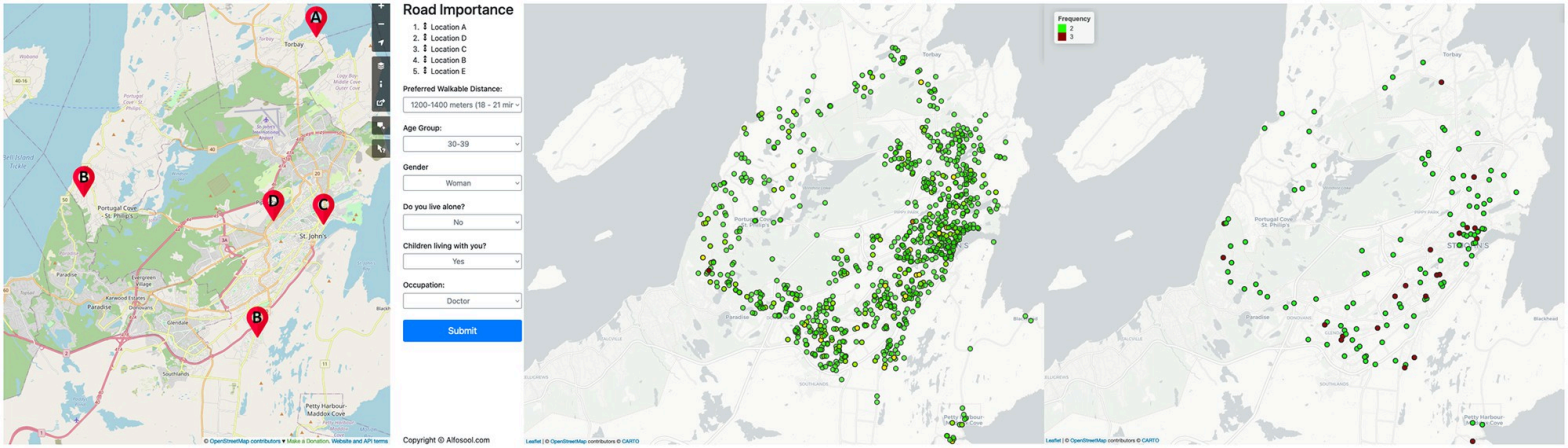
ALF–Score online crowd–sourcing platform. Our interactive web–based data collection platform on left (map generated by OpenStreetMap, screenshot taken from alfosool.com) has been deployed with road data from various cities in Canada. Displayed here shows the city of St. John’s, NL. Total 1050 (center^1^) rankings were received from participants showing a well distributed data collection. Some locations were ranked multiple times (right^2^) by the same or various participants. We can observe in our collected data the maximum number of conflicts is 3 per location with a very little occurrence. (Maps ^1^ and ^2^ are generated through RStudio [31] Version 1.2 using mapview package from rstudio.com with OpenStreetMap used as its base map).

To further expand on our crowd–sourcing platform and the collected data which play crucial roles in providing the required user opinion, we will first explore how the data collection takes place followed by why we chose our approach. To grant ourselves the ability to collect accurate user data and ensure data completeness while reducing bias, we developed a web interface that is capable of collecting various information from volunteer users such as: walkability order of 5 randomly selected locations (relative ranking among the 5 locations), users preferred walkable distance, their age group, gender, if users Live alone, if users have children, their occupation, and a few other browser agent details that are publicly available (such as users browser type, operating system, etc.). The web interface shown of Fig 3, left, displays an interactive map with 5 randomly selected locations marked as {*A*, *B*, *C*, *D*, *E*}. Every time the web page is reloaded, 5 randomly selected locations are chosen from a given geographic area. The coverage is tied to a pool of nodes derived from our map database, specifically the road network structure of the region.

The randomization method is introduced to ensure an evenly distributed coverage of node labels and user opinions across the given geographical area. Users have the ability to reorder the ranking of the 5 locations by moving them up or down based on how they perceive the relative walkability of the shown locations among each set of 5 locations. This approach reduces a potential cognitive challenge and allows users to use their intellect and knowledge of the city while exploring a diverse set of potential locations in the region. The relative rankings are then submitted as groups of 5 locations, to the server for downstream processing. Given the random nature of the web tool, the group of 5 locations provided by the web tool can overlap across different users or even different submissions of the same user. On one hand, the overlap provides common references among submission groups, but on the other hand, the submission groups will likely accumulate perceived conflicts across their relative rankings. We can observe from Fig 3, right, all the locations with 2 or more associated submissions, leading to conflicts.

The decision to collect the data labels as a combination of relative rankings and not absolute scores (eg. ranking each 5 locations between a fixed range such as 0–100 where the same rank may apply to other locations) was made to reduce bias, conflict and variability in the data while incentivizing the participants to make a precise decision to determine which of the given 5 locations would be most or least walkable and order the locations as deemed appropriate. While absolute scores are easier to use, each individual may have a completely different rational when it comes to why a location has been ranked the way it is, for example why 65 and not 40. Utilizing relative rankings takes that factor out and allows us to focus on the most important factor: whether location *A* is more walkable than location *B* for a given individual. This way, there will be a concise approach to determine each individual’s perception towards walkability when comparing different points together over the responses of other users. Conflicts are unavoidable and lead to inconsistencies among user opinions. The challenge here is to balance the opinions collected from users to yield a walkability metric that is agreeable to most users. User opinions collected maintain a unique form observing only relative orders within each submission of 5 locations and therefore do not represent the global view among the overall data. Due to this and the nature of the conflict resolution, the problem remains in the NP–complete space, and we therefore need to device a new approach to handle conflicts and represent user opinions within a global perspective.

### Generalized Linear Extension of Partial Orders—GLEPO

Generalized Linear Extension of Partial Orders or GLEPO, is an algorithm we have devised specifically to process this approach of user opinion label collection. GLEPO produces a generalized list of all user opinions in total order and absolute ranks to represent relative ranks of small submissions, within a global observation of the overall data. This conversion is especially important as the users rank locations within small groups of 5 locations. The order of places in these small groups is unique and only relative within the same group among the 5 locations. Rankings $o_{i}^{r}$ between 1 − 5 that are relative only within their own set of 5 nodes where *i* ranges between 1 and the total number of nodes in a single submission (5). The $o_{i}^{r}$ relative rankings similarly apply to all user submissions within *S*. Each *S*_*j*_ submission maintains 5 nodes holding a unique rank between 1 − 5 where *j* ranges between 1 and the total number of submissions. $o_{i}^{r}$ is completely localized at this stage and in no relationship with nodes from other submissions. In order to correctly utilize user data in conjunction with their submitted rankings and find the missing link between various submissions, we need to evaluate and establish a unified relationship between all nodes within user submitted data.

The first step of GLEPO is to group the submissions into multiple lists *S*_*j*_ containing 5 nodes {*v*_1_, *v*_2_, *v*_3_, *v*_4_, *v*_5_} each. The next step is to detect anchor nodes. Anchor nodes are defined as those commonly and naturally occurring nodes that repeat in various submissions. For instance, if we have submission *x* = {*n*_1_, *n*_2_, *n*_3_, *n*_4_, *n*_5_} and submission *y* = {*n*_6_, *n*_7_, *n*_3_, *n*_8_, *n*_9_}, node *n*_3_ would be considered as an anchor node defining a connection between submissions *x* and *y*. GLEPO algorithm uses anchor nodes to define connections between two or more submissions with a concept that if there’s an anchor node between two submissions, using the position of the anchor node(s) we are able to narrow down an approximate positioning between the nodes of the connected submissions. Submissions may have none, 1 or more anchor nodes. For instance, in the example above, since *n*_3_ is the anchor node and it also happens to be in the centre of both submissions *x* and *y*, we can deduce that {*n*_1_, *n*_2_} must fall before {*n*_8_, *n*_9_} and similarly {*n*_6_, *n*_7_} must fall before {*n*_4_, *n*_5_}.

Our main routine (Algorithm 1) iterates through the user data to form one possible variation of a newly sorted global list. When an anchor node is detected, the submission containing the anchor node is passed along to *addToSorted*() function to be evaluated and to decide where the node entries within that submission are placed. This process helps appropriately address the anchor nodes’ associated relationship with regards to nodes’ ranks, while sorting the entries based on their user–submitted order. However, in the case where multiple anchor nodes are detected, special conditions may be invoked within *addToSorted*() function that determines the best course of action.

GLEPO’s sorting subroutine (Algorithm 2) parses the passed submission where one (or more) anchor node(s) has been detected. The core of this algorithm revolves around determining the number of anchor nodes, their positions within the current submission and associated position in the partially sorted list, and invoking the appropriate case associated to each condition. There are 4 main conditions two of which are associated with the detection of only a single anchor node positioned either at the beginning or the end of the submission in process. Third condition is applied when the anchor node is not at the beginning or the end of the submission in process and applies to two cases: 1) if only a single anchor is detected, or 2) multiple anchors detected but the anchor in process is the last anchor. The final case accounts for all other conditions. Each case determines the segment or segments of the submission in process that requires insertion into the global list. These segment is then passed to another function to insertion into the list. This algorithm essentially loops over all anchor nodes comparing each with the appropriate condition to determine a list of potential insertion locations. This list is then passed to the randomized insertion subroutine for a more balanced insertion within the accepted positions. It is possible the list of possible insertion locations may contain either a single location or multiple suitable locations.

**Algorithm 1** GLEPO: Main Routine

**Input:** User data organized by submission: *subs_grouped*

**Output:** List of sorted user data entries: *sorted_entries*

1: initialize *sorted_entries* as an empty list

2: initial randomization by shuffling *subs_grouped*

3: **for** every submission group *sub_g* in *subs_grouped* **do**

4:  **if** *sorted_entries* is empty **then**

5:   **for** every submission *sub* in *sub_g* **do**

6:    append *sub* to *sorted_entries*

7:  **else**

8:   **for** every submission *sub* in *sub_g* **do**

9:    **if** submission node exists in *sorted_entries* **then**

10:     add submission to list of anchor nodes.

11:   **if** anchor node(s) detected **then**

12:    pass *sub_g* and anchor list to *addToSorted()* function

13:   **else**

14:    pass *sub_g* to *FindVLink()* function

15: return *sorted_entries* list

Due to the structure of the data input and the nature of the algorithm, different outcome is expected after every run of the algorithm if any variation in the order the input is fed into the algorithm is detected. For example, the order of submissions and the nodes can affect the decision making processes of the algorithm when conflicts are detected. Due to this, we introduced 2 randomization components into the process. The first randomization component is applied within our main subroutine (Algorithm 1) which randomly shuffles the order of appearance in all submission input. The second randomization component can be observed being called from our sorting subroutine (Algorithm 2) invoking our randomized insertion subroutine (Algorithm 3) which randomizes the locations nodes can be inserted into the global list by determining all appropriate positions and randomly choosing one to insert the node in process. Algorithm 3 receives a list of nodes to insert, a list of currently sorted nodes as well as a list of acceptable insertion positions. For every node to be inserted, a randomly selected location is selected from the list of all acceptable insertion positions and the node in process is inserted there. All used locations are then removed from the list. Minimum and maximum insertion are indices indicative of a segment of the sorted list that is suitable to insert the selected node in process. With the randomization introduced, the entire GLEPO process requires to run multiple iterations until the global list starts to converge. This convergence is associated with the order each node appears within the global list over each run. The main process averages the order for each single node over the course of all iterations and determines the final order in which each node falls under after multiple iterations. Due to this approach, it is possible, multiple nodes may have similar ranking which is the expected results. We tested the algorithm over multiple numbers of iterations such as 10, 30, 50, 80 and 100 iterations. We found that around 30 iterations and onward the generated global list starts to converge and yield a stable result.

**Algorithm 2** GLEPO: Sorting Subroutine

**Input:** Submission group *sub_g* alongside its list of anchor node(s)

**Output:** Updates: *sorted_entries*

1: set current pointer to 0

2: **for** every anchor node **do**

3:  **if** it’s the only anchor and is positioned at the beginning of *sub_g* **then**

4:   set segment to all 4 elements to its right

5:   pass segment to *RandomizeInsertion()* function

6:  **else if** it’s the only anchor and is positioned at the end of *sub_g* **then**

7:   set segment to all 4 elements to its left

8:   pass segment to *RandomizeInsertion()* function

9:  **else if** it’s the last anchor but positioned in the middle of *sub_g* **then**

10:   set left segment to all elements to its left

11:   set right segment to all elements to its right

12:   pass left segment to *RandomizeInsertion()* function

13:   pass right segment to *RandomizeInsertion()* function

14:   update current pointer to current anchor position + 1

15:  **else**

16:   set segment to all elements between current pointer and anchor’s position

17:   pass segment to *RandomizeInsertion()* function

18:   update current pointer to current anchor position + 1

19: return *sorted_entries* list

**Fig 4:**
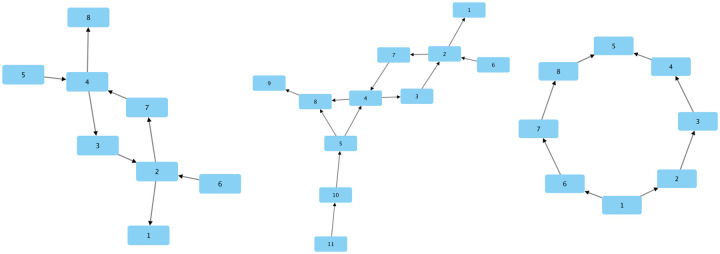
Three examples of some of the artificial networks created to represent various possible scenarios of user submissions and conflicts. Left: graph of 2 submissions (each with 5 nodes) containing 2 anchor nodes ‘4’ and ‘2’ forming a loop. Center: graph of 3 submissions with 4 anchor nodes. Right: graph of 2 submissions containing 2 anchor nodes ‘1’ and ‘5’ falling on two extreme ends. (Figures drawn by the authors).

An important step here is to ensure GLEPO is capable of handling special cases, no matter how rare their occurrence may be. To this end, we created various artificial networks that presented different scenarios within the user submission data set. Each of these artificial networks was converted into a graph and processed by GLEPO and the resulting output was tested and verified to ensure the algorithm always returns results with the least amount of possible inconsistency (defined later in this chapter). In Fig 4 we demonstrate 3 artificial networks used for verification of GLEPO’s accuracy and consistency. We can observe there are a few possible (and correct) outcomes for each of these graphs. For example, in Fig 4 right, node 5 is the highest ranked node among the two submissions whereas node 1 is the lowest ranked node among the two. Therefore, based on users opinion and the limited information given by only these two submissions, we know node 5 should be ranked the highest and node 1 the lowest. In this example, all other nodes could form number of different orders as long as they remain true to their respective submission. To name a few possible outcome: [1, 2, 3, 4, 6, 7, 8, 5], [1, 6, 7, 8, 2, 3, 4, 5], [1, 2, 6, 3, 7, 4, 8, 5]. We can observe node 1 is maintaining the least rank while node 5 is the highest in all generated outcomes. We can further observe nodes from each submission always follow their original order of: [2, 3, 4] and [6, 7, 8]. It is important to reiterate the significance of the randomization component and how it will help form a uniformed distribution of nodes into positions within GLEPO’s output that are most appropriate.

To ensure the sorted list remains true and consistent to the user submitted rankings we examine its consistency to that of the original user data. Each user submission consists of 5 road nodes 〈*v*_1_, *v*_2_, *v*_3_, *v*_4_, *v*_5_〉, where each node *v*_*i*_ (*i* = 1, 2, 3, 4, 5) has a ranking *r* denoted $o_{i}^{r}$, (“order”) between 1 and 5. The rank $o_{i}^{r}$ defines users’ assigned order to each node with respect to the other 4 nodes within that submission. Now when the nodes from all submissions are sorted using the GLEPO algorithm into a unified global list, the most natural approach to check for any inconsistencies is to verify the order of all the original user submissions with that of the global list. If the order of the 5 nodes in each of the original submission remains intact to that appearing within the global list, then the nodes within that submission are considered fully consistent to that of the original user’s opinion. If the order differs however, all out–of–order nodes are counted and amounted to an inconsistency value. This is done by a pair–wise comparison of each submission appearing in two separate lists: 1) a list in order submitted by the user, 2) a list in order appearing in the sorted list.

Based on our earlier definition of anchor nodes, submissions containing one or more anchor nodes become anchored. In contrast, we define those submissions without an anchor node as constraint–free submissions. Based on this definition, we can observe at least two possible challenges with GLEPO pertaining to how well–anchored the submissions are with respect to user data: 1) over constraining, 2) under constraining. When adding new nodes to the general list, over constraining occurs when excessive conflicts and inconsistencies are detected, through anchor nodes. For example, when majority of the submissions have multiple conflicts with multiple submissions, we are faced with over constraining. With more crowd–sourced user opinion, comes more potential conflicts and disagreement. A natural solution to this is the embedded randomization of GLEPO over multiple iterations to ensure a well distribution within the input list and node insertion has been applied by GLEPO to produce the resulting global list.

**Algorithm 3** GLEPO: Randomized Insertion Subroutine

**Input:** Current list of sorted entries, minimum insertion range, maximum insertion range, list of elements to insert

**Output:** Updates: *sorted_entries* by randomly inserting new elements within the given range

1: **if** the size of the list of elements to insert is less than the range between min and max **then**

2:  for the size of the list of elements to insert, generate random numbers within min and max range

3:  sort the generated random indexes

4:  **for** a range between 0 and the size of the list of elements to insert **do**

5:   insert an element of the list into *sorted_entries* at position within the generated numbers

6: **else**

7:  for the size of the range between min and max, generate random numbers within the size of the list of elements to insert

8: sort the generated random indexes

9:  **for** a range between 0 and the size of the range between min and max **do**

10:   insert an element of *sorted_entries* at selected position into the list of elements to insert at position within the generated numbers

11:   replace elements of *sorted_entries* within min and max range with updated list of elements to insert

12: return *sorted_entries* list

Under constraining on the other hand occurs when there’s an insufficient number of connections (or anchors) among user submissions. This lack in the number of anchored submissions leads to an inability to establish an accurate association among various submissions. These associations enable the algorithm to present the data within a global list that reflects the global view of all users with respect to each other. Within our crowd–sourced data, we have observed that majority of user submissions do not have an anchor node and are constraint–free, hence leading to under constraining. Two main approaches are considered when addressing this challenge, both of which are complimentary and can be done concurrently. First, collection of more user data. Second, through creating virtual anchor nodes, or virtual links. Virtual link is defined as an artificial connection between two or more submissions created specifically to associate constraint–free submissions that do not have any anchor node associated to those submissions already processed by GLEPO. Virtual link utilizes geographical proximity to establish a virtual connection between a single submission and the partially complete global list, by determining the closest nodes (in terms of physical distance) among the two. Algorithm 4 shows our approach to creating virtual links. This subroutine is called through our main routine whenever a submission without an anchor node is detected. The improved computational complexities for various algorithms in the pipeline are highlighted in Table 1.

**Table 1:** Computational complexity of various algorithms in the pipeline.

Algorithm	Big–O
Distance Matrix Measurement	*O*(*n*^2^)
Sorting Subroutine	*O*(*n*)
Randomized Insertion Subroutine	*O*(*n*)
Virtual Link Creator	*O*(*n*)
GLEPO	*O*(*n*^2^)

**Algorithm 4** GLEPO: Virtual Link Subroutine

**Input:** Current list of sorted entries, submission group *sub_g*, distance matrix of all nodes

**Output:** Create a virtual link for the given *sub_g* and updates *sorted_entries* accordingly

1: set the desired threshold—maximum distance to consider a virtual link

2: initialize main shortest distance to a very high number

3:  **for** every element in *sorted_entries* **do**

4:   find element’s distance to all 5 nodes within *sub_g*

5:   **if** shortest distance among the 5 is shorter than main shortest distance **then**

6:   set main shortest distance to new distance

7:   set main shortest node to associated node

8: **if** main shortest distance *<* threshold **then**

9:  treat main shortest node as an anchor node

10:  pass *sub_g* and main shortest node to *addToSorted()* function

11: **else**

12:  pass the entire *sub_g* to *RandomizeInsertion()* function

13: return *sorted_entries* list

### Active Living Feature Score—ALF–Score

The Active Living Feature Score or ALF–Score, is a novel measure of walkability that aims to create a better walkability scoring system capable of generating walkability scores at a higher spatial resolution (point–level) that are representative of user opinion while more informative to most users. ALF–Score takes a user–centric approach instead of the traditional researcher–centred approach. ALF–Score aims to better utilize road network structure and characteristics and derive node features that are defined by various road characteristics and their direct and indirect associations that can consider how the underlying road structure can influence neighbourhood and city structure and walkability. ALF–Score employs user–based parameters such as user opinion of walkability, to better represent user perception and provide user–derived scores of what users would perceive as various degrees of walkability.

At the core of ALF–Score’s pipeline lies various machine learning approaches and methods with the goal of training machine–learned models that are capable of estimating numeric values defining how walkable specific points on the road are, based on various feature combinations that include road network, road embedding and POI features. This approach would help estimate walkability scores based on node characteristics. The input maintains a combination of various features derived from road structure and POIs generated by different methods while user opinion acts as training labels. The ideal outcome is to avoid calculating walkability score individually for every single point on the road, and have the ability to accurately estimate point–specific scores, from only a small sample of crowd–sourced labelled data. The output of the machine learning pipeline would be a trained model and the output of the model will be walkability score predictions for given nodes. The score is defined as how walkable users may find the selected points, an output that will be normalized to 0~100.

When it comes to the machine learning pipeline, we are working with a node regression problem as we seek to estimate continuous numerical output (walkability scores) based on numerical features (some converted from other types such as categorical, ordinal, etc.). To address a regression problem, there are many possible approaches such as linear regression, random forest, support vector regression (SVR), and multi–layer perceptron neural network (MLP) which are explored in this research. The prediction input variables describing data features are represented as: {*x*_1_, *x*_2_, *x*_3_, …, *x*_*n*_}, while the output is represented by *y*, respectively. We use labelled sets such as {features, label}: {*x*, *y*} to train the model. The expectation from trained models is to produce a prediction where given an unlabelled set such as {features,?}: {*x*,?} where? would be replaced with *y*′ prediction. The models, which are defined by internal parameters learned through the process, map unlabelled example sets to predicted value *y*′. Since some of the features are not numerical (eg. categorical or ordinal), we have used one hot encoding, where applicable, to convert all features into appropriate numerical entries. The developed machine learning pipeline has five primary components: 1) GLEPO, 2) consistency measure and verification, 3) Feature selection, 4) Model training, 5) Model validation.

Output of the model, walkability score for any given point within the data set, is robust and refined with much higher spatial resolution, compared to other walkability measures. The nature of the output and their predictive structure can be used to create interactive interfaces that allow users visually view walkability scores for any selected areas with as high as point–level resolution. Furthermore, due to the nature of the model, there is be no need to calculate the walkability score for the entire network. Predictions for small subsets or even the entire network, as needed, can be made available in fraction of the time required to calculate the walkability scores using traditional methods.

Our desired walkability metric is a global scalar which is consistent across all road nodes that have user input. But it is also coherent across all road nodes with and without user input labels. Our walkability function is defined as *w*: *V* ⇒ *R*^+^ where *V* is a set of vertices and *R* is a set of user rankings. The performance metric of *w* is based on consistency with user rankings *R*. Specifically, the performance metric considers user ranking *r* ∈ *R* involving a set of 5 nodes {*v*_1_, *v*_2_, *v*_3_, *v*_4_, *v*_5_}. Ranking *r* gives each node *v*_*i*_ (*i* = 1, 2, 3, 4, 5) a rank denoted $o_{i}^{r}$, (i.e. the “order” of node *v*_*i*_ as they appear in user ranking.) Similarly, walkability *w* would also imply for *v*_*i*_ (*i* = 1, 2, 3, 4, 5) a rank denoted $o_{i}^{w}$, (i.e. the “order” of node *v*_*i*_ as suggested by *w*.) Furthermore, the loss function of *w* with respect to *r*, or the “inconsistency” between *r* and *w*, can be defined as

$$L\left(r,w\right)=\sum_{i=1}^{5}\left|o_{i}^{r}-o_{i}^{w}\right|$$

while the loss function with respect to *R* is aggregate of *L*(*r*, *w*):

$$L\left(R,w\right)=\sum_{j=1}^{l}L\left(r_{j},w\right)$$

That is, the total amount of inconsistency of *w* as compared to all user–provided rankings. The aggregate function can take other forms too, such as mean or multiplication. There can also be other ways to define such an inconsistency measure, e.g. *l*_2_-norm or “out–of–order” counts, or Kendall rank correlation coefficient [36]. The goal is to determine the most appropriate *w* that minimizes *L*(*R*, *w*), the amount of inconsistency between *w* and *R*. A conversion from “relative” ranking associated within a small localized set of nodes provided by a single user to a “global” list of scores which represents relativity among all users and their provided opinions within the network with as little discrepancy among *R* as possible is a crucial step.

We have applied various machine learning techniques and have compared their accuracy based on various feature set combinations. The goal is to find the most suitable technique as well as feature combination that produce the most appropriate models predicting accurate walkability scores. The techniques that were used in both supervised and semi–supervised environments were: 1) random forest, 2) linear regression, 3) decision tree, 4) support vector regression (SVR), 5) gradient boosting, 6) polynomial features (a non–linear approach), 7) lasso CV and 8) multi–layer perceptron neural network (MLP). Feature combinations used are: 1) only POI features, 2) only network features (centrality measures), 3) only road embedding features, 4) POI + road network features, 5) POI + road embedding features, 6) road network + road embedding features, 7) all features. In this work various hyper–parameters were experimented with. For instance, MLP models were structured as sequential models with multiple dense layers as hidden layers with *ReLU* activation function in conjunction with *mean_absolute_error* loss function and *adam* optimizer while the number of dense layers ranged between 2 and 12. Furthermore, best decision tree models were trained on *best* splitter, and a maximum depth varying between 2 and none while best gradient boosting models were trained with a maximum depth of 10, 100 boosting stages, and a learning rate ranging between 0.1 and 1.0. In our SVM models the default kernel type of *rbf* was used alongside regularization parameter set to 1.0, epsilon of 0.2 and shrinking heuristic while linear regression models had normalization and intercept parameters enabled. In random forest training the number of trees in the forest was set to 100 with no maximum depth set.

It is important to point out that ALF–Score is a novel and unique approach with no similar measures for direct comparison. Our success metric when it comes to determining the accuracy of our models, is based on how close the results are to user opinions and the general knowledge of the area, based on 2 main approaches: 1) using validation and test sets to verify the models, 2) visual inspection and verification based on local knowledge of various cities. As with most machine learning approaches, various challenges are observed especially when it comes to keeping our models as generalized as possible. For example, lack of sufficient data, biased in data collection or processing, and incorrectly labelled data are some of the challenges that can lead to modelling errors. Overfitting and underfitting, are also two very common modelling errors that occur when the model’s main function is too closely fit to the data (too well–learned) for the case of overfitting, or when the function does not capture the prominent patterns in the data (not enough learning) in the case of underfitting. Overfitting leads to high accuracy in training results (previously seen data), but significantly lower accuracy when applied to new (unseen) data. Underfitting on the other hand, leads to unpredictable outputs. In both cases, low generalization of the models lead to unreliable predictions. Furthermore, one of the prominent challenges faced in this research is that the crowd–sourced data is relatively small. Two directly associated issues are: 1) potential bias in lack of variety in participating user groups, 2) lack of appropriate coverage when it comes to relative walkability scores associated to various road network structures.

There are many ways to address these challenges. One of which that we have used in this research is data split. The core concept of data split is to separate the labelled data into two data sets: 1) training set, 2) testing set. For example, in a 80–20 percent split, 80 percent of the labelled data is assigned to training set while the remaining 20 percent form a testing set. Typically a 70–30 or 80–20 percent split is commonly seen, but we have also experimented with various other variations such as 90–10 and 60–40. The labels from the test set are never shown to the algorithms while training the models. The prediction results are then compared to the actual results to determine a base line on the accuracy and performance of various models. Furthermore, a well–balanced split can make a huge difference when it comes to a well generalized model. For instance, when working with a small set of data, if too much or too little is assigned to either the training or the test set, the model may not perform well due to lack of observable patterns. Additionally, Mean Absolute Error (MAE) and Root Mean Squared Error (RMSE) were used to measure each model’s error. Mean Absolute Error (MAE) is defined as $MAE=\frac{\sum_{i=1}^{n}|y_{i}-x_{i}|}{n}$ where *x*_*i*_ is the actual value, *y*_*i*_ is the prediction, and *n* is the total number of data points. Root Mean Squared Error (RMSE) on the other hand is defined as $RMSE=\sqrt{\frac{\sum_{i=1}^{n}(x_{i}-y_{i}{)}^{2}}{n}}$. *K*–fold cross validation [37] is used as yet another method to address this challenge. *K*–fold cross validation is typically done by splitting the data set into *k* smaller sets. One set is selected as the test data set, and the remaining sets are combined as one training data set. Once the model is built, it is then evaluated on the test data set and the procedure repeats for every set in *k* to ensure the entire data set is fully utilized towards building and evaluating the models.

## Results

In total, 666 features are used to train various models, with some models trained with a specific subset of these features, to examine the resulting accuracies based on various features. We use the following input parameters:

1. Road network data *G* = (*V*, *E*) which covers city of St. John’s, NL. *G* is connected, undirected and unweighted. |*V*| = 5,341 nodes and |*E*| = 6,835 edges. Road node features, a mix of numeri ordinal, and categorical data. 1) POI Features with 530 features. These features are derived from 8 separate OSM categories with 53 total sub keys. Each single key contributes to 10 features. Each of which reflects on a specific geometrical distance range showing whether that particular key falls within the associated distance of any point within the network. The geometrical distance ranges from 200m to 2,000m with 200m increments. These 530 features are extended to every node on the road network. 2) Network Features which contributes 8 features, namely: Betweenness centrality, Closeness centrality, clustering coefficient, degree, eccentricity, neighborhood connectivity, stress, topological coefficient. 3) Network Embedding with 128 features using node2vec [38].
2. Crowd–sourced data. There are 35 unique users with |*S*| = 210, where *S* represents all user submissions. Each *S*_*j*_ where (*j* = 1, 2, …, |*S*|) represents a user submission. Each *S*_*j*_ user submission consists of 5 road nodes 〈*v*_1_, *v*_2_, *v*_3_, *v*_4_, *v*_5_〉. We chose to collect rankings of 5 nodes per submission with careful consideration. With small number of places (i.e. 2 locations) there is not enough relativity among submitted nodes. With too many locations (i.e. 10 locations) we risk posing cognitive challenges when users decide rankings between many locations. To ensure sufficient relativity yet avoid cognitive challenges, there needs to be enough location points to create a balanced association among various nodes. There are total of 1,050 user–submitted locations among their relative ranks. From these 1,050 locations, there are 852 unique locations. leaving 198 “anchor” locations (defined in Methods section) appearing in two or more submissions. Each road node *v*_*i*_ (*i* = 1, 2, …, 5) has a unique relative ranking $r_{v_{i}}$ between {1, 2, …, 5}. The order of $r_{v_{i}}$, the relative ranking, is of utmost importance as it defines how users perceive the walkability of each *v*_*i*_ with respect to one another.

Using our Generalized Linear Extension of Partial Orders or GLEPO algorithm, we were able to successfully convert user opinion relative among groups of 5 locations into globally relative scores (among all submissions) with only a small inconsistency (see Method for description). GLEPO maintains a high consistency of 98.24% throughout the conversion. Furthermore, after numerous variations and experimentation, we have determined that the best results were produced using the fully randomized version of the algorithm with at least 30 iterations. Virtual link was enabled when best results were produced. Virtual link is a method we developed that establishes associations between user submissions based on their geographical distance (explained further in Methods).

**Fig 5:**
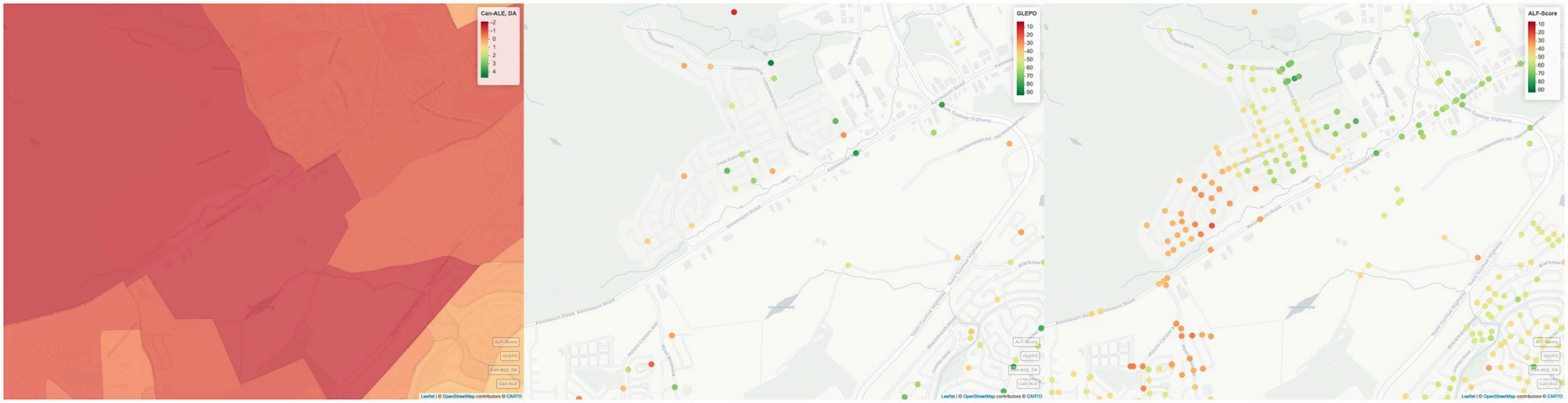
Comparison between GLEPO, ALF–Score and Can–ALE for Great Eastern Ave. in St. John’s, NL. Left: Can–ALE, Center: GLEPO, Right: ALF–Score. A score variation ranging between 16–81 is observed within GLEPO which provides a user–level insight about how users actually perceive their neighbourhood in their own opinion, as opposed to what Can–ALE suggests of a neighbourhood to its users. Furthermore, we can observe a variation ranging between 20–70 in ALF–Score which provides a high spatial resolution rankings that are well refined and specific to each point as opposed to a single score for an entire area as observed in Can–ALE. Note: Can–ALE colors are slightly dimmed due to the adjusted opacity/transparency to better visualize the street overlay. (Maps generated through RStudio [31] Version 1.2 using mapview package from rstudio.com with OpenStreetMap used as its base map).

When compared to Can–ALE, a clear variation is observed, especially when it comes to producing fine–tuned high spatial resolution ground–truth. In Fig 5, we can observe while Can-ALE (left) is dedicating a low walkability score to large regions, users have different varying opinions (center) ranging from low walkable to high walkable scores, for different points on the road of the same region. GLEPO therefore provides a more representative and high spatial resolution ground–truth. This paper is not intended to imply that Can–ALE is incorrect, but rather pointing out some of its limitations and shortcomings due to its characteristics. Since Can–ALE is area–based its scores provide a low resolution coverage. Since there is no use of user opinion in its pipeline, Can–ALE scores are not representative of users’ opinion. As observed in GLEPO’s result, not only our volunteer participants have a difference of opinion with that of Can–ALE’s provided walkability scores, but there’s also a difference in opinion among participants themselves which opens up the possibility for future research on applying GLEPO and ALF–Score to different sub groups of users.

Although GLEPO’s results provide an overall well–represented user opinion baseline to walkability scores while establishing a ground–truth for ALF–Score’s machine learning pipeline as its *y* label vector, GLEPO’s main contributions may be extended to vast range of researches such as enabling accurate use of crowd–sourced data while reducing bias as a result of difference in opinion. Fig 5 further expands on how predicted walkability scores generated by ALF–Score (right) compares with that of Can–ALE (left), and how ALF–Score is able to provide high spatial resolution walkability scores as opposed to existing area–based walkability measures. We found a high variation in ALF–Score walkability rankings while observing an even distribution withing the selected region. As locations get closer to the Kelsey Drive region they are marked as more walkable by ALF–Score. (There are numerous amenities within the vicinity of Kelsey Drive.)

We used GLEPO’s output to train our supervised machine learning models as a regression problem on our 666 features iterating over multiple variations of data combination subsets. Six major techniques were used, namely: 1) Random Forest Regression, 2) Linear Regression, 3) Support Vector Regression (SVR), 4) Gradient Boosting, 5) Decision Tree Regression, and 6) Multi layer Preceptions (MLP). We found that Random Forest outperforms other techniques in terms of performance and accuracy by achieving a top prediction accuracy of 87.49% using all features. In Table 2 we take a closer look at all feature combinations used in our experiments as well as their best achieved accuracy. All accuracy values are representative of the best recorded accuracy over numerous runs. We observed models trained on only POI features performed relatively similar to that of those models trained on POI plus network features and POI plus road embedding. However, models trained on network features combined with network embedding appear to perform better than those using POI previously motioned (with the exception of linear regression). Highest accuracy was observed when all features were used together. This presents a convincing argument in contrast to that of some other walkability measures’ hypothesis, which assume high importance towards POI features. Furthermore, it also conveys an important message that road network structure plays a crucial role when it comes to measuring walkability. The improvement in accuracy observed after the addition of POI features to the network and road embedding features contributes to the complimentary positions POI and road network structure have with respect to one another, where combined can provide a more in–depth understanding of our surroundings.

**Table 2:** Exploration of various machine learning techniques and feature combinations with their top performing accuracy.

Technique	POI	POI + Network	POI + Embedding	Network + Embedding	All
**Random Forest**	77.36	79.49	79.40	81.07	**87.49**
**Linear Regression**	51.15	54.57	61.74	30.86	**72.21**
**SVM**	66.47	68.50	66.35	75.38	**76.36**
**Gradient Boosting**	60.30	59.16	58.17	68.75	**68.78**
**Decision Tree**	65.33	67.52	64.98	75.89	**76.35**
**MLP**	69.86	70.94	73.18	74.63	**79.87**

Although deep neural network techniques such as MLP are generally expected to provide more accurate results, they require a tremendous amount of data. As it was observed, this could be an issue when dealing with only a small set of data like the one used in this research. It is believed that as more user data is accumulated, MLP’s performance will improve.

**Fig 6:**
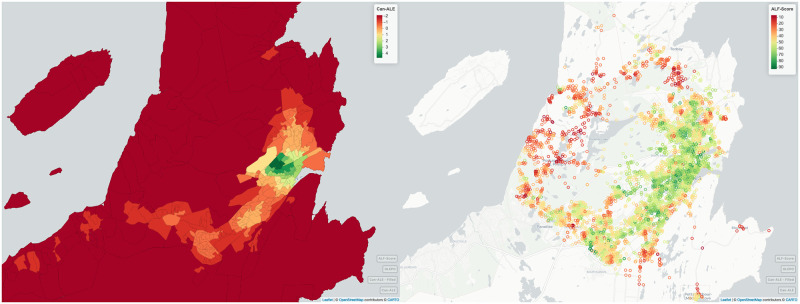
A comparison between Can–ALE and ALF–Score for the city of St. John’s, NL. Can–ALE (left) and ALF–Score (right). Dark green is most walkable, dark red is least walkable. Can–ALE’s scores may be rendered useless due to its over generalization. (Maps generated through RStudio [31] Version 1.2 using mapview package from rstudio.com with OpenStreetMap used as its base map).

In Fig 6 we can observe how ALF–Score (right) compares with one of the most prominent walkability scores namely Can–ALE (left) for the walkability of the city of St. John’s, NL (Canada). In the walkability produced by Can–ALE, we can observe various regional “blocks” (DAs) where nodes within them carry the exact same score. Some of these regions may cover much larger geographical areas, whereas other regions may cover a higher population density in a smaller geographical area. This significantly reduces the accuracy, relevancy and spatial resolution of the scores when using Can–ALE. As observed in Fig 6 right, this issue is no longer the case with ALF–Score walkability ranks as they are much more refined, with clear variability among different nodes’ scores, and relevant when compared to Can–ALE data with significantly higher spatial resolution, right down to every point on the road. Furthermore, ALF–Score walkability rankings are representative of user opinion with higher accuracy associated to users’ perception of walkability in different regions. We were able to successfully verify the accuracy of our predicted ALF–Score rankings based on local knowledge of the city of St. John’s.

**Fig 7:**
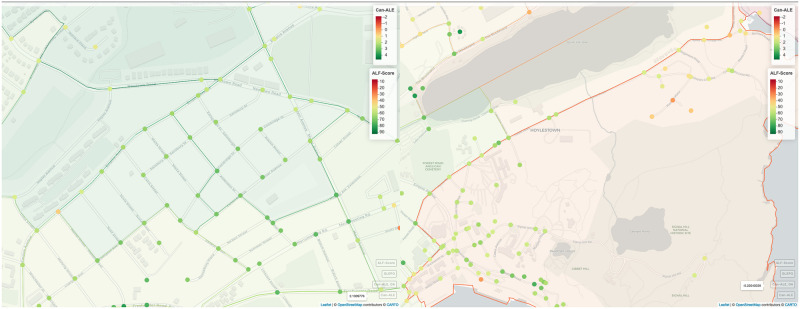
Examples of where ALF–Score and Can–ALE do and do not agree. Each DA polygon is represented by a single Can–ALE value while each circle represents an ALF–Score. Left: Downtown St. John’s, NL, shows a strong agreement among the two measures. Right: Signal Hill region, St. John’s, NL, shows a strong disagreement between the two measures. Note: Can–ALE is represented as an overlay with a small opacity/alpha and is visualized with a significantly lighter colors due to this transparency. Legends and border lines represent the actual colors. (Maps generated through RStudio [31] Version 1.2 using mapview package from rstudio.com with OpenStreetMap used as its base map).

In addition, we have observed that typically areas with greater population density are assigned with higher Can–ALE scores. This may not always be how users actually perceive walkability. In Fig 7 which represents two examples where ALF–Score and Can–ALE do (right) and do not agree (left), we found Can–ALE fail to identify regions such as local in–city parks and trails as walkable areas as observed in Fig 7 left. The Signal Hill area is a well–known and commonly visited area by the local community especially hikers, runners, joggers and families. There are numerous amenities nearby such as convenience stores, restaurants, coffee shops and gas stations and there are multiple bus stops. Yet, the area was considered as not walkable by Can–ALE. The analysis suggests that Can–ALE’s approach is missing out on some of the important features and area characteristics that people consider important such as being near to ponds, trails, and national monuments. We found that ALF–Score is able to do a much better user representation and provide more in–depth rankings for this area as well as many others in our experiments by better capturing various characteristics while associating their importance based on user opinion.

## Discussion and conclusion

An important factor about this research is its interdisciplinary contributions, specifically in the fields of Computer Science and Public Health. Interdisciplinary researchers are crucial and typically provide practical applications to many important and immediate challenges at hand. Day–to–day users are among the intended users of ALF–Score, but they are not the only intended users. We hope this research can be used as a tool that allows researchers in the public health sector, specially the epidemiologist, to have a better understanding of how positioning, accessibility and mobility, specifically walkability, is associated with health outcomes, including physical activity and obesity. Technology is advancing every day and what better use of it but to improve people’s lives and health, and we genuinely believe these kind of practical interdisciplinary research can truly and positively impact the world.

The purpose of this research was to fill in the gap and address some of the most prominent challenges observed in existing walkability measures by introducing a novel approach to measure walkability score with high spatial resolution, precision, accuracy and efficiency. In this section, we will discuss some of the main contributions of this research to computer science and public health communities.

We believe a strong indicator of walkability can be derived from road importance which requires extensive utilization of road network structure. However, there’s very little use of road network structure, if at all, in most existing walkability measures. This lack leads to a significant gap in utilizing one of the most important factors of walkability and hence lower accuracy. Although an important consideration is that road network structure can be quite large and creating a graph structure while calculating graph–based features (such as centralities and network embeddings) pose various challenges (which we have addressed in a previous publication [4]), our walkability results using road network structure show an important improvement, especially when combined with user opinion in conjunction with our machine learning pipeline. Road network structure provides crucial information about neighbourhoods and cities. Furthermore, road network structure is also a conduit to propagate the importance of POIs. We further improved existing walkability measures by incorporating user opinion and perception as one of the crucial contributing components in the pipeline. Existing walkability measures lack user opinion and we believe walkability scores generated should consider public opinion.

Use of crowd–sourced data in ALF–Score opens the door to many new possibilities such as building a platform to provide personalized walkability based on each individual’s profile. This will not be possible without the use of user opinion. Users’ observations and demographics can be included into building personalized models that are able to identify user patterns and generate models to best fit individual user profile.

Collection of user crowd–sourced data is expensive, time consuming, limited and resource intensive. It is difficult to recruit volunteer participants who have the knowledge of the city and are willing to help with providing their personal walkability scores that would amount to sufficient data to be used as ground truth. It is also important to consider that walkability is subjective, so people will have different opinions. Specifically, when it comes to walkability scores, each user is entitled to their personal opinion and everyone may and likely will have a different opinion on how walkable they may consider specific locations. This expected variation can potentially lead to noticeable inconsistencies within the user labels. We addressed this challenge in two phases by 1) developing our own web–based crowd–sourcing tool to maximize data calibration and accuracy, 2) using GLEPO to further process the collected data to ensure fair distribution within localized relative scores and accurate globalized representation among all users. Accuracy of ground–truth data is crucial as it is representative of the population through only a small sample size and we believe as more data is collected, accuracy will be improved.

The concept and processes behind our data collection method and GLEPO can be applied to many other fields when it comes to utilizing opinion–based data as it allows for collection of small and relative data sets leading to reduced bias and presumptive measures, such as defining a hard–coded range for walkability. For instance, if users were to rank walkability score for locations, by assigning a number between 0–100, locations will get varying scores associated to them by different users. This assignment is dependant on how user define the numerical range and how that aligns, or otherwise, with that of the researcher. Utilizing relativity as opposed to absolute scores, allows for reduction in the imposed cognitive challenge of assigning a representative score for each location, and instead, users utilize relative ordering by indicating if they consider a location more or less walkable than another location without assigning a fixed numerical value. GLEPO will then take the responsibility of globalizing the relative ranks to reflect how they would rank within all other user submissions. This gives researchers the flexibility to maintain their own rational while preserving user opinion integrity.

To tie in with the previous points, since ALF–Score lies on crowd–source data, in the initial stages, only a small amount of data is available to represent two key components: 1) user opinion representative of a much larger population 2) road nodes representative of much larger geographical regions and road networks. Based on our experimentation and results, our pipeline has proven to accurately generate walkability scores for various cities using only a small sample data.

Furthermore, we were able to significantly improve the spatial resolution of walkability, by utilizing road network, user opinion and machine learning approaches that help generate point–level walkability scores of all nodes within a given road network, as opposed to area–based approach found in some existing measures. This higher spatial resolution of walkability provides a much needed depth for analysis and other researches that require fine–tuned, refined and specific scores for various locations that may fall within close proximity and within the same DA.

The approaches in this research are not fixed. They are designed with the intention of full flexibility. Characteristic features may be replaced. Network data can be changed. Ground truth can be representative of another value and be generated via other methods. These possibilities open the door to building a flexible metric capable of wide variety of fine–tuning that could lead to new sets of resulting output. ALF–Score can be adjusted to generate bikeability or transit–accessibility scores and much more. Its predictive approach has made it possible to successfully achieve its goals while only requiring a small set of data making it a very user–friendly and research–friendly tool. Furthermore, as various components of this research produce reliable inputs to the model training pipeline, the overall pipeline can be used in conjunction with various future extensions to further improve ALF–Score and to build more specific and more robust walkability measuring tools. ALF–Score has paved the path to other future works such as full personalization of walkability and transferable walkability models that we will introduce in our future works.
